# Tripleosteotomie bei Morbus Perthes

**DOI:** 10.1007/s00064-022-00784-5

**Published:** 2022-09-16

**Authors:** Kai Ziebarth, Nadine Kaiser, Theddy Slongo

**Affiliations:** Kinderchirurgische Universitätsklinik, Abteilung Kinderorthopädie, Inselspital Bern, Freiburgstr., 3010 Bern, Schweiz

**Keywords:** Morbus Perthes, Beckenosteotomie, Tripleosteotomie, Femurkopf, Containment, Perthes disease, Pelvic osteotomy, Triple osteotomy, Containment, Femoral head

## Abstract

**Operationsziel:**

Durch die Osteotomie des Ischium‑, Pubis- und Iliumknochens kann das Acetabulum über den Hüftkopf geschwenkt werden, sodass der meist anterolateral vorstehende Anteil des Hüftkopfes wieder überdacht wird. Das Ziel ist der Erhalt des Containments der Hüfte, hiermit ist die Wiederherstellung der Kongruenz zwischen dem lateralisierten Hüftkopf und dem Acetabulum gemeint. Das Acetabulum wirkt so als eine Art Schablone für den Hüftkopf, um eine möglichst sphärische Ausheilung des Hüftkopfes zu erreichen.

**Indikationen:**

Schwere Morbus-Perthes-Erkrankung mit radiologisch sichtbarer Lateralisation des Hüftkopfes und Head-at-risk-Zeichen. Voraussetzung ist, dass der Hüftkopf sich konzentrisch reponieren lässt (Abduktionsaufnahme oder Arthrographie).

**Kontraindikationen:**

„Hinged abduction“. Keine konzentrische Reposition des Hüftkopfes möglich.

**Operationstechnik:**

Arthrographie des Hüftgelenkes zur Bestätigung der Operationsindikation. Darstellung und Osteotomie des Ischiums über einen modifizierten Ludloff-Zugang, Osteotomie des Iliums und Pubis über einen modifizierten Smith-Petersen-Zugang. Verbesserung der Hüftkopfüberdachung durch Schwenken des Acetabulums über den Hüftkopf. Fixation des azetabulären Fragmentes mit Vollgewinde-Kirschner-Drähten oder 3,5-mm-Kortikalisschrauben.

**Weiterbehandlung:**

Mobilisation an Gehstöcken (kleine Kinder im Rollstuhl). Abstellen des Fußes erlaubt. Teilbelastung für 4 bis 6 Wochen (je nach Alter des Patienten). Danach bei im Röntgen guten Konsolidationszeichen Belastungsaufbau innerhalb 1 bis 2 Wochen.

**Ergebnisse:**

Sehr gute Ergebnisse hinsichtlich Operationstechnik und Ausheilung in der eigenen Klinik. In einer eigenen noch nicht veröffentlichten Studie mit einem durchschnittlichen Untersuchungszeitraum von 5 Jahren zeigten sich bei 30 Patienten sehr gute klinische und radiologische Ergebnisse nach Tripleosteotomie bei Morbus Perthes.

## Abstract (en)

**Objective:**

Osteotomy of iliac–pubic and ischial bone in order to cover the lateralized femoral head with the acetabulum. With the restoration of the containment of the hip joint, the acetabulum functions as a template for the femoral head, thus, allowing it to keep its sphericity during the vulnerable stages of Legg–Calve–Perthes disease.

**Indications:**

Lateralized femoral head in severe Legg–Calve–Perthes disease and visible head at risk signs on the radiographs. Prerequisite is possible concentric reduction of the femoral head (confirmed by preoperative abduction radiograph or arthrography).

**Contraindications:**

Hinged abduction. Impossible concentric reduction of the femoral head.

**Surgical technique:**

Hip arthrography to confirm the indication of the triple pelvic osteotomy is recommended. Osteotomy of the ischial bone by a modified Ludloff approach. Osteotomy of pubic and iliac bone by anterior approach (Smith Peterson/bikini incision). Turning the acetabulum over the femoral head allows improvement of the containment of the hip. Fixation of the acetabulum with fully threaded Kirschner wires or 3.5 mm cortical screws.

**Postoperative management:**

Touch-down weight bearing with crutches (wheelchair in younger children) for 4–6 weeks depending on the age of the child. After radiologic evidence of consolidation, transition to full-weight bearing within 1–2 weeks.

**Results:**

Promising results in our own practice. Good functional and radiological results in a to-date unpublished study of 30 patients with Legg–Calve–Perthes disease after an average 5‑year follow-up.

## Vorbemerkungen

Die Behandlung des Morbus Perthes richtet sich nach dem Ausmaß der Erkrankung. Ist der Hüftkopf nur in einem kleineren Areal betroffen (Herring A) [1], bleibt die Kongruenz des Gelenkes erhalten (Containment), und konservative Maßnahmen wie temporäre Entlastung des Beines und Physiotherapie sowie Sportkarenz für hüftbelastende Tätigkeiten sind ausreichend [2].

**Figure 1:**
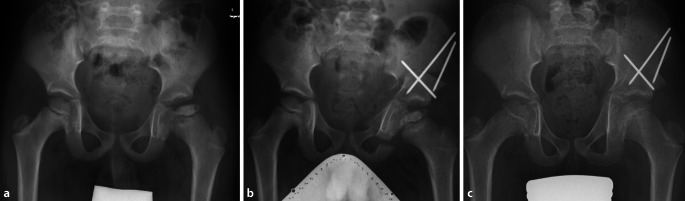

Kommt es aber zu einem Befall eines signifikanten Anteiles des Hüftkopfes (Herring B, C), besteht die Gefahr der Lateralisation des Hüftkopfes (s. Abb. 1 und 2). Hierdurch geht das Containment verloren, dies bedeutet, dass die Kongruenz zwischen Acetabulum und Hüftkopf nicht mehr erhalten ist. In dieser Situation kann es zu einer asphärischen Ausheilung des Hüftkopfes kommen. Dies ist häufig mit einer schlechten Funktion der Hüfte und der Gefahr der Früharthrose vergesellschaftet. Gleichzeitig kann bei schweren Perthes-Erkrankungen eine sekundäre Hüftdysplasie mit steil aufsteigendem lateralem Acetabulum resultieren [3].

**Figure 2:**
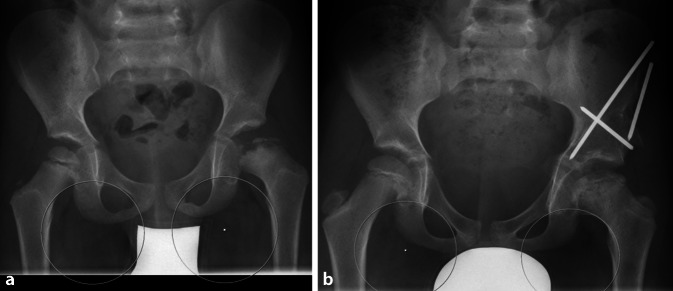

Gemäß Literatur ist der Verlauf des Morbus Perthes bei Kindern < 6 Jahren als prognostisch besser einzuschätzen, weswegen von einigen Autoren in dieser Altersstufe die Indikation zur konservativen Behandlung großzügiger gehandhabt wird [4]. Jedoch zeigen sich auch bei sehr jungen Kindern schwerwiegende Verläufe mit Lateralisation des Hüftkopfes oder bereits sichtbarer sekundärer Hüftdysplasie, hervorgerufen durch den Druck des kranialisierten Hüftkopfes auf das laterale Acetabulum.

Allgemein akzeptiert wird in der neueren Literatur, dass eine Intervention möglichst zu einem frühen Zeitpunkt der Erkrankung (Fragmentationsstadium) durchgeführt werden sollte, um durch die Kongruenz (Containment) des Gelenkes ein sphärisches Ausheilen des Hüftkopfes zu ermöglichen.

Ob die korrigierende Operation auf femoraler oder azetabulärer Seite durchgeführt werden sollte, wird kontrovers diskutiert. Es zeigt sich jedoch zunehmend ein Trend zur azetabulären Korrektur [5, 6].

Die Tripleosteotomie (Durchtrennung aller 3 Anteile des Beckens zur Reorientierung des Acetabulums) ist zur Behandlung der kindlichen Hüftdysplasie in vielen verschiedenen Techniken (Tönnis, Steel, Le Coeur, Sutherland) bereits publiziert worden. Die hier beschriebene Technik entspricht der Berner „Modifikation“ der Tripleosteotomietechnik. Ziel ist, die Osteotomien so nah wie möglich am Gelenk durchzuführen und durch einen abgewinkelten Iliumschnitt den Knochenkontakt und damit die Knochenstabilität-/heilung zu verbessern. Der Vorteil der Tripleosteotomie besteht darin, den Femurkopf wieder im Acetabulum zu zentrieren und damit das Containment des Gelenkes zu erhalten. Durch diesen Zustand kann der Hüftkopf im Verlauf der Morbus-Perthes-Erkrankung sphärisch ausheilen, wobei das Acetabulum als natürliche Schablone für den Hüftkopf wirkt.

Die Biomechanik des Hüftgelenkes wird bei der Tripleosteotomie im Gegensatz zu femoralen Eingriffen nicht negativ beeinträchtigt. Ein bekanntes Problem z. B. bei der Varusosteotomie des Femurs ist, dass die Hüftabduktoren geschwächt werden. Zusätzlich wird die bereits durch den Morbus Perthes vorbestehende Beinverkürzung durch eine varisierende Osteotomie noch verstärkt [2].

## Operationsprinzip und -ziel

Der lateralisierte Anteil des Femurkopfes soll nach der Triple OT so überdacht werden, dass es während der Reparationsphase zu einer möglichst sphärischen Ausheilung des Hüftkopfes kommt.

## Vorteile

- Korrekte Zentrierung des Hüftkopfes durch Schwenken des mobilen azetabulären Fragmentes
- Keine Beeinträchtigung der Hüftabduktoren
- Keine femorale Fehlstellung
- Keine weitere Beeinträchtigung der Beinlänge

## Nachteile

- Anspruchsvolle Operation
- Gegebenenfalls mehr Blutverlust durch Beckenosteotomie als z. B. durch Femurosteotomie
- 2 Hautinzisionen (alternativ wird auch eine Technik mit Single-Inzision beschrieben, [7])

## Indikationen

- Morbus Perthes mit Verlust des Containments (Zentrierung des Hüftkopfes)
- Hüftdysplasie

## Kontraindikationen

- „Hinged abduction“
- Keine konzentrische Reposition des Hüftkopfes im Acetabulum möglich

## Patientenaufklärung

- Allgemeine Operationsrisiken: Infektion, Blutung, Gefäß‑/Nervenverletzungen, Narkoserisiko
- Verletzung des Ischiasnervs bei Ischiumosteotomie
- Pseudarthrose des Ischium durch zu starke Korrektur oder Interposition von Muskulatur
- Verzögerte Heilung oder Pseudarthrose im Bereich der 3 Osteotomien durch knöcherne Diastase oder Interposition von Muskulatur
- Überkorrektur, Unterkorrektur oder falsche Korrektur
- Intraartikuläre Implantatlage (KD, Schrauben)
- 2 Operationsnarben (medial und ventral)
- Gefäßverletzungen
  A. circumflexa femoris medialis (Ischiumosteotomie)
  A. obturatoria (Ischiumosteotomie, Pubisosteotomie)
  A. iliaca externa (Iliumosteotomie)
  A. iliace interna oder Truncus posterior (Iliumosteotomie)
  A. pudenda interna (Iliumosteotomie)
- N. cutaneus femoris lateralis (Smith-Petersen Zugang)
  N. ischiadicus (Ischiumosteotomie)
  N. obturatorius (Ischiumosteotomie, Pubisosteotomie)

## Operationsvorbereitungen

- Analyse der radiologischen Diagnostik:
  - Becken a.-p./Lauenstein oder „cross table axial“
  - Abduktionsaufnahme: Zentrierung Hüftkopf (Wiederherstellung der Shenton-Menard-Linie), azetabuläre Überdachung, „hinged abduction“
- MRI betroffene Hüfte: Ausmaß des Morbus Perthes. Größe/Sphärizität des Femurkopfes, Gelenkschäden, Konsequenzen s. Artikel Femurkopfreduktion im selben Heft

## Instrumentarium

- Hohmann-Haken (8 + 16 mm)
- An der Spitze abgerundete Hohmann-Haken (sog. weiche Eva-Haken)
- Umgekehrte Eva-Haken
- Langenbeck-Haken
- 3,0–4,0 mm Schanz-Schrauben (Joy stick)
- Vollgewinde-Kirschner-Drähte (Fixation)
- Bildwandler

## Anästhesie und Lagerung

- Intubationsnarkose inklusive vollständiger Muskelrelaxation
- Rückenlagerung auf röntgendurchlässigem Tisch
- Gewichtsadaptierte Antibiotikaprophylaxe (2.-Generation-Cephalosporin), 30 min präoperativ intravenös, dann 8‑stündlich bis 48 h postoperativ

## Operationstechnik

(Abb. 3, 4, 5, 6, 7, 8, 9, 10, 11, 12, 13)

**Figure 3:**
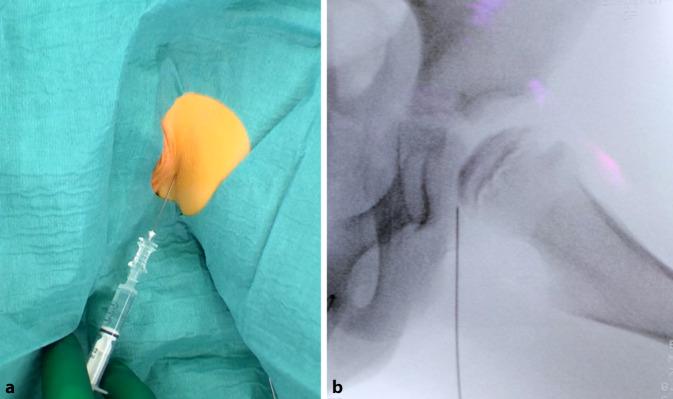

**Figure 4:**
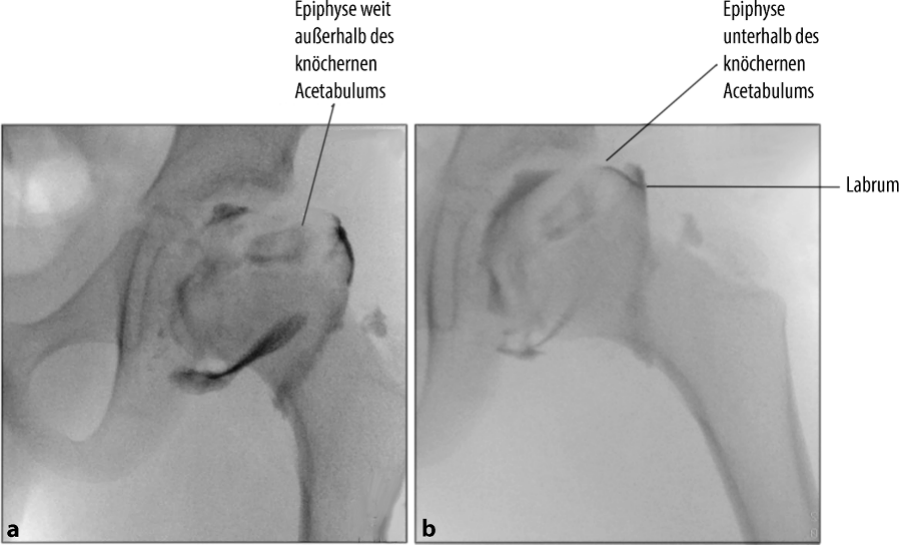

**Figure 5:**
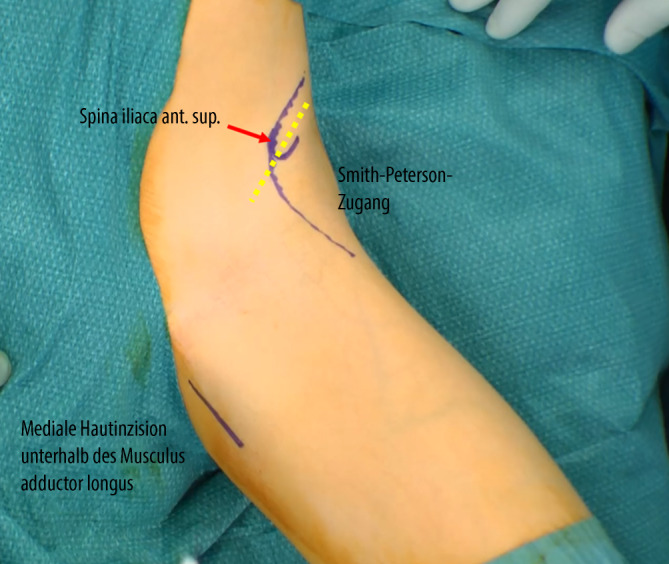

**Figure 6:**
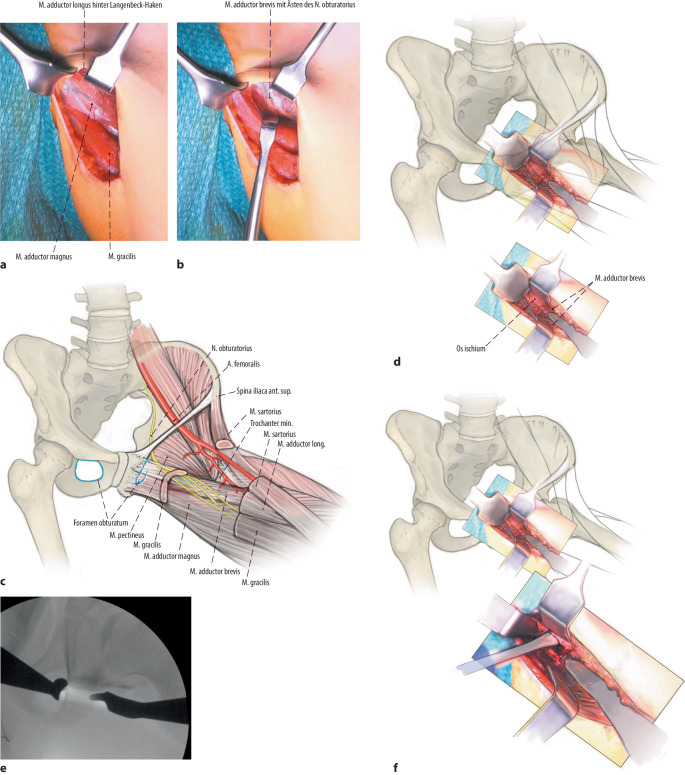

**Figure 7:**
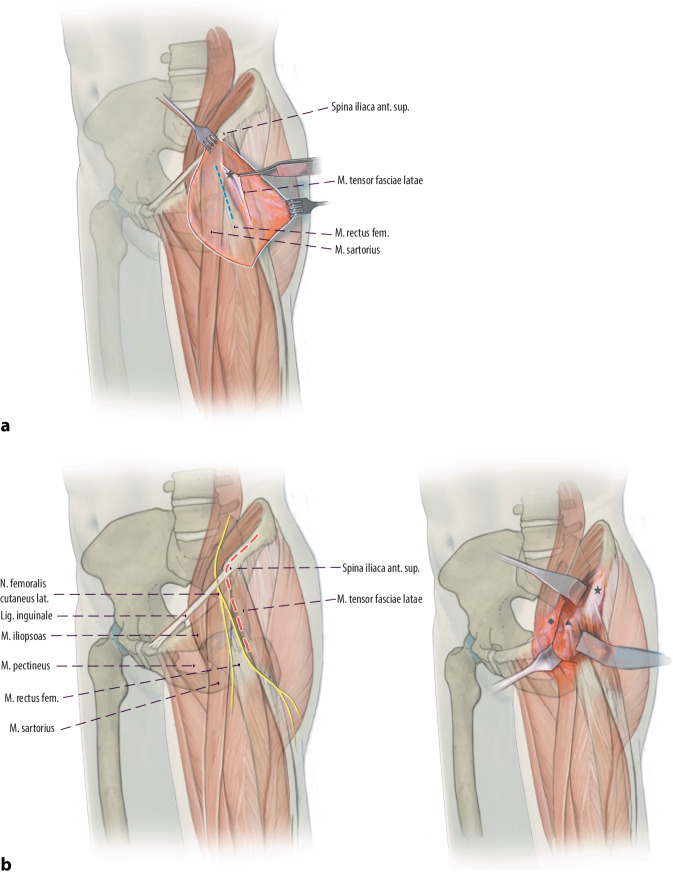

**Figure 8:**
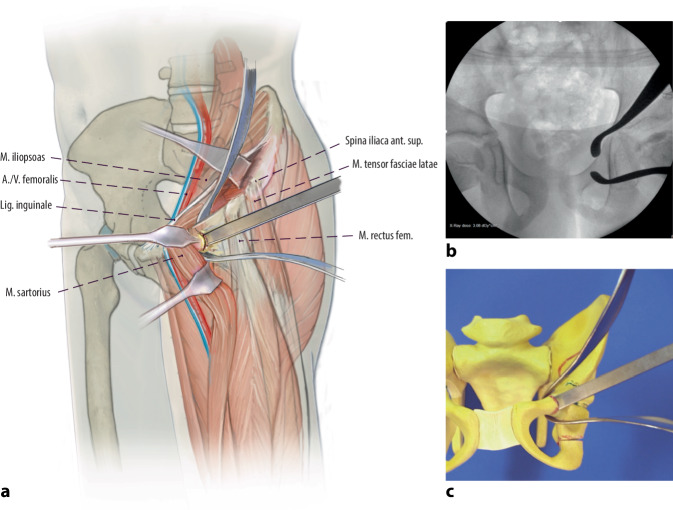

**Figure 9:**
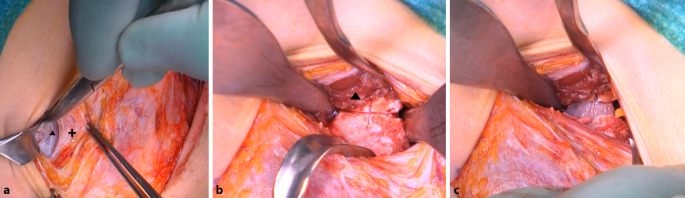

**Figure 10:**
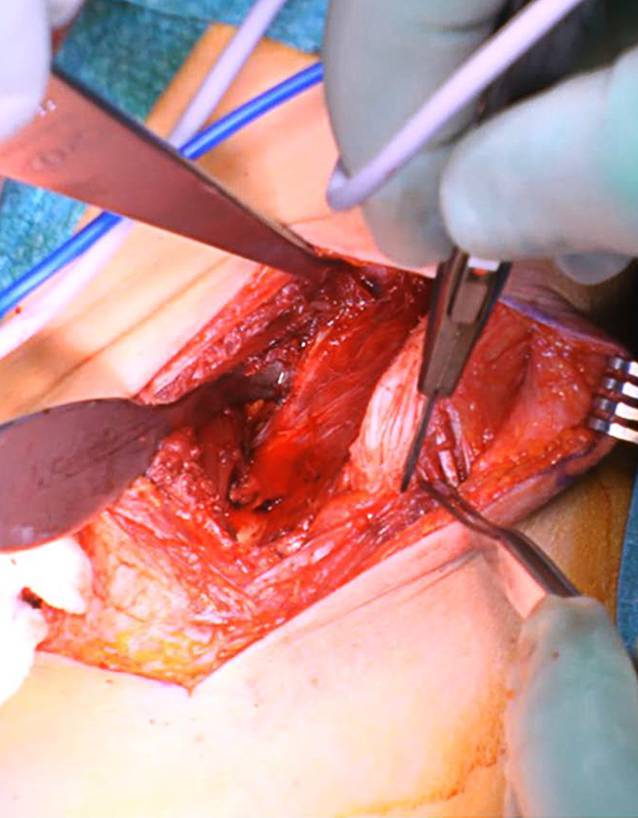

**Figure 11:**
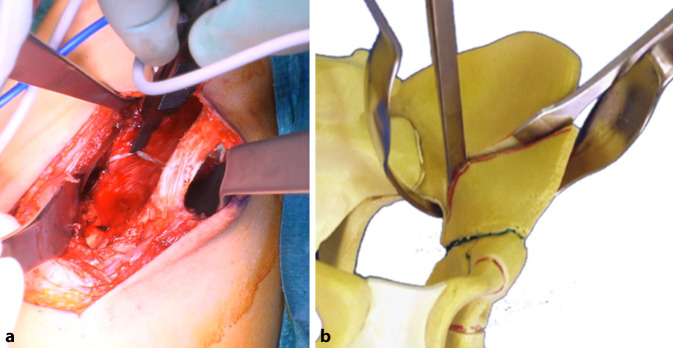

**Figure 12:**
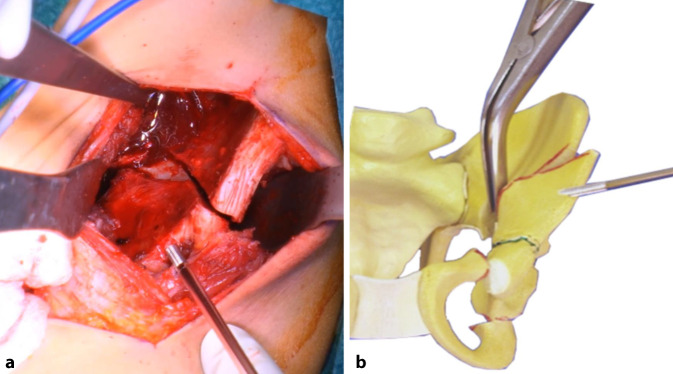

**Figure 13:**
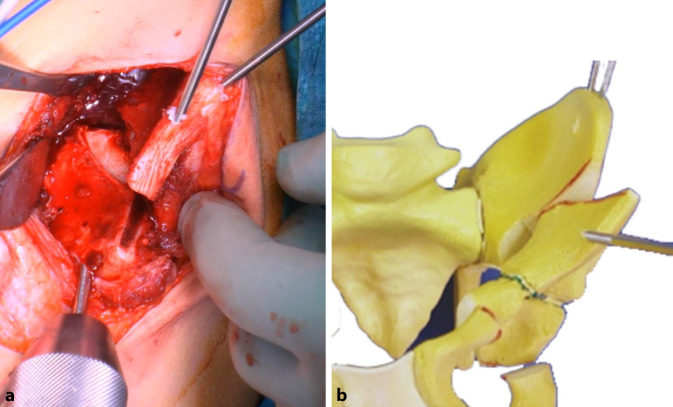

## Postoperative Behandlung

- Teilbelastung an Gehstöcken „touch down“ für 4 bis 6 Wochen (je nach Alter des Patienten Mobilisation im Rollstuhl), dann Kontrolle mit Röntgen
- Nach 6 Wochen bei guter Konsolidation Übergang zur vollen Belastung je nach Verlauf des Morbus Perthes
- Langzeitkontrollen bis Ausheilung des Morbus Perthes

## Fehler, Gefahren, Komplikationen

- Schlechte Indikation („hinged abduction“), hier wäre eine Hüftkopfreduktion zu erwägen mit oder ohne Beckenosteotomie (s. Artikel in diesem Heft)
- Gefäßverletzung A. femoralis. Sofortige Gefäßnaht/Rekonstruktion (sehr selten, keine klaren Angaben in der Literatur)
- Gefäßverletzung A. obturatoria oder Corona mortis. Korrekte Blutstillung intraoperativ
- Verletzung des N. obturatorius, N. cutaneus femoris lateralis, klinischen Kontrolle, in der Regel keine Intervention notwendig
- Verletzung des N. ischiadicus, wenn postoperativ evident, Kontrolle ob Traktion vs. Durchtrennung. Allenfalls Revisionsoperation mit Freilegung und/oder Rekonstruktion der Nerven bei Durchtrennung. Bei Traktionsschaden abwarten
- Inkorrekte Reposition (keine freie Mobilisation des azetabulären Fragmentes wegen periostaler Adhäsionen, inkomplette Osteotomien), Revision mit Komplettierung der Osteotomien und erneuter Reposition
- Instabile Fixation, schnellstmögliche Revision
- Pseudoarthrose (meist Os ischium): eher bei ausgeprägten Korrekturen wie z. B. neuromuskulären Dysplasien. Bei Therapie des Morbus Perthes nicht zu erwarten, da Korrektur nicht so ausgeprägt

## Ergebnisse

In einer noch nicht veröffentlichen Studie mit 30 retrospektiv analysierten Patienten (Durchschnittliches Follow-up 5 Jahre) mit Herring-B/C- und -C-Hüften und einem Durchschnittsalter von 7,5 Jahren zum Zeitpunkt der Operation zeigten sich exzellente Ergebnisse hinsichtlich Extrusionsindex (präoperativ 32,9 ± 6,2, postoperativ 13 ± 4,5) und azetabulärer Winkel (LCE Winkel präoperativ 13,9 ± 10,7, postoperativ 37 ± 8,1, gesunde Seite 33,2 ± 6,7). Die Lebensqualität gemessen mit I‑Hot, WOMAC- und Harris Hip-Score zeigten ebenfalls sehr gute Ergebnisse. Revisionen mussten in diesem Patientenkollektiv nicht durchgeführt werden. Nerven oder Gefäßschäden traten ebenfalls nicht auf. Vukasinovic [5] berichtet bereits 2009 in seiner Studie über ähnlich gute Ergebnisse der Behandlung von Morbus-Perthes-Patienten mit einer Tripleosteotomie, allerdings in einer etwas anderen Technik als der hier beschriebenen. In einer Studie von Stepanovic [6] wird die Revisionsrate nach Tripleosteotomie mit 20 % angegeben, wobei die Revisionen weniger aus direkten Komplikationen, sondern wegen aufgetretener mechanischer Probleme, wie z. B. femoroazetabulärem Impingement bei Überkorrektur, oder erneuter Subluxation des Hüftkopfes resultierten. Signifikante Nerven-Gefäß-Schäden oder auch Infektionen wurden in beiden Studien nicht beschrieben und sind auch in der Literatur äußerst selten erwähnt. In einer Arbeit über Komplikationen bei Beckenosteotomien von Renner [8] wurde eine mögliche Gefäßschädigung bei einer Tönnis-Tripleosteotomie von 2–3 % angegeben und Nervenläsionen zwischen 6 und 32 % beschrieben. Diese hohen Zahlen, v. a. der Nervenläsionen, können bei der von uns beschriebenen Operationstechnik nicht bestätigt werden. In unserem Patientenkollektiv, in dem die Tripleosteotomie angewendet wird (nicht nur Morbus-Perthes-Patienten) kommt es selten zu einer transienten Hyposensibilität des N. cutaneus femoris lateralis am ehesten durch intraoperative Traktion durch Weichteilhaken. Diese ist für den Patient aber meistens nicht sehr störend und gibt sich im Verlauf der Zeit wieder von alleine. Eine partielle Fußheberschwäche nach schwierigen Tripleosteotomien ist ebenso bekannt, tritt jedoch eher bei komplizierten Fällen (schwere Hüftdysplasie), oder Revisionsoperationen und nicht bei der Therapie des Morbus Perthes auf. Eine vollständige Durchtrennung des Ischiasnervs, welcher eine Revisionsoperation erforderte, ist nach Tripleosteotomie in unserer Klinik bisher nicht aufgetreten.

Die Retroversion oder generelle Fehlpositionierung des Acetabulums ist ein bekanntes Problem. Aufgrund des jungen Alters der Patienten ist anhand des Röntgenbildes die Orientierung des Acetabulums schwer zu beurteilen, da die Pfannenränder noch nicht ossifiziert sind (erst vollständige Ossifikation mit 12 bis 13 Jahren). Somit muss man sich nach indirekten anatomischen Landmarken richten (Spina ischiadica, Shenton Linie), um eine korrekte Positionierung des Azetabulum zu erzielen und Unter-Überkorrekturen zu vermeiden. Ebenso empfiehlt es sich, die Funktion der Hüfte intraoperativ vor und nach der Korrektur zu prüfen. Im Zweifel kann im Anschluss an die Korrektur eine erneute Arthrographie helfen, die Version und Inklination des Acetabulums zu überprüfen.

In einer Studie von Hosalkar [9] konnte gezeigt werden, dass nach ausgeprägter Korrektur durch die Tripleosteotomie bei Patienten mit offener Y‑Fuge ein gewisses Remodelling noch festzustellen ist.

In der eigenen Klinik ist die Tripleosteotomie das Mittel der Wahl im Falle einer chirurgischen Behandlung des Morbus Perthes und hat die varisierende Femurosteotomie aus den in dem Artikel erwähnten Gründen fast vollständig verdrängt.
